# Cytotoxicity, Post-Treatment Recovery, and Selectivity Analysis of Naturally Occurring Podophyllotoxins from *Bursera fagaroides* var. *fagaroides* on Breast Cancer Cell Lines

**DOI:** 10.3390/molecules21081013

**Published:** 2016-08-04

**Authors:** Omar Aristeo Peña-Morán, María Luisa Villarreal, Laura Álvarez-Berber, Angélica Meneses-Acosta, Verónica Rodríguez-López

**Affiliations:** 1 Facultad de Farmacia, Universidad Autónoma del Estado de Morelos, Cuernavaca, Morelos 62209, Mexico; pmoa_ff@uaem.mx (O.A.P.-M.); angelica_meneses@uaem.mx (A.M.-A.); 2 Centro de Investigación en Biotecnología, Universidad Autónoma del Estado de Morelos, Cuernavaca, Morelos 62209, Mexico; luisav@uaem.mx; 3 Centro de Investigaciones Químicas, Universidad Autónoma del Estado de Morelos, Cuernavaca, Morelos 62209, Mexico; lalvarez@ciq.uaem.mx

**Keywords:** selective-index, *Bursera fagaroides*, podophyllotoxins

## Abstract

Despite prevention and treatment options, breast cancer (BC) has become one of the most important issues in the present day. Therefore, the need for more specific and efficient compounds remains paramount. We evaluated four previously isolated aryltetralin lignans: 5′-demethoxy-β-peltatin-A-methylether (**1**), acetylpodophyllotoxin (**2**), 5′-demethoxydeoxypodophyllotoxin (**3**), and 7′,8′-dehydroacetylpodophyllotoxin (**4**) for cytotoxicity, clonogenicity, and selectivity against three BC cell lines: MCF-7, MDA-MB-231, and BT-549, as well as the non-tumorigenic mammary epithelial cell line MCF-10A. Cytotoxicity was evaluated after 72 h of treatment, and clonogenicity was determined at 72 h post-treatment; experiments were performed using the sulforhodamine B staining assay. Selective-index (SI) was calculated by comparing pure compound IC_50_ values in MCF-10A cell line against the IC_50_ of the same compound in cancer cell lines. Structural similarities among lignans and controls (podophyllotoxin and etoposide) were analyzed using the Tanimoto coefficient (Tc). Lignans were cytotoxic against all tested cell lines (0.011–7.22 µM) and clonogenicity testing showed a dose-dependent cytocidality for all lignans (≥0.08 µg/mL); compounds **2** and **3** were more potent (14.1 and 7.6 respectively) than etoposide in BT-549 cell line, while compound **2** displayed selectivity (SI = 28.17) in BT-549 cell line. Tc values of lignans suggested a greater similarity with podophyllotoxin structure.

## 1. Introduction

Cancer is a group of diseases responsible for 8.2 million deaths (13% of all deaths worldwide), according to data collected by the World Health Organization (WHO) in 2012. Unfortunately, the WHO has estimated that this number will increase to 30 million in the next 20 years. Among different cancers, breast cancer has become one of the most important issues, as 25% of female cancer patients suffer from it [1]. This is considered a public health problem because of the high costs of treatment. Therefore, even though several prevention and treatment options are available, the search for more specific and efficient compounds remains paramount in order to avoid adverse effects caused by typical treatments [2]. Breast cancers are routinely classified by stage, pathology, grade, and by the expression of estrogen receptor (ER), progesterone receptor (PgR), or human epidermal growth factor receptor (Her2/neu) [3].

Some of the chemical compounds used for cancer treatment are natural molecules isolated from plants or semisynthetic drugs, natural compounds chemically treated and modified to achieve a better activity or/and less toxicity. Podophyllotoxin (POD), a lignan compound initially isolated from *Podophyllum peltatum* [4], has been used as a folk medicine; owing to its severe toxic side effects, this compound is limited to topical applications [5,6,7]. However, in the last century, different modifications on its chemical structure resulted in the development of the epipodophyllotoxin semisynthetic drug “etoposide” (also called VP-16) [8]. This compound is less toxic than POD and it is used as an anticancer drug against several cancers, such as lung, ovarian, and testicular cancer, as well as lymphoma [9]. By binding to β-tubulin, the POD mechanism of action is through the inhibition of the mitotic spindle during mitosis, resulting in cell cycle arrest in the G_2_/M phase and consequent programmed cell death [10,11,12]. On the other hand, VP-16 has a different mechanism of action associated with the inhibition of DNA-topoisomerase II. By forming a ternary complex with the enzyme [13], it prevents the re-ligation of DNA, inducing a pre-mitotic blockage in the late S or early G_2_ stages [14,15].

**Figure 1:**
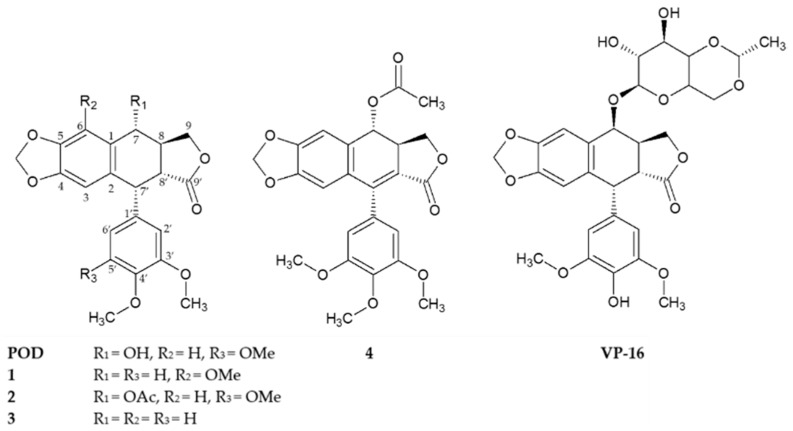
Chemical structures of compounds **1**–**4** isolated from the stem bark of *Bursera fagaroides* var. *fagaroides*, and the positives controls podophyllotoxin (POD) and etoposide (VP-16).

Structurally, podophyllotoxins consist of a five rings system (A to E) including a dioxol ring (A), tetralin rings (B and C), γ-lactone ring (D), and an aromatic ring (E) of α configuration (Figure 1) [10]. Several studies have shown that only the A and E rings are essential for podophyllotoxins activity and that aromatization of the C ring leads to a loss of activity [5]. VP-16, unlike POD, undergoes a stereotransformation at C-7 from an α to a β configuration (epipodophyllotoxins), and a demethylation at C-4′ in the E ring [14,16]. These main structural changes indicate how the action mechanism, from a cytotoxic compound that interacts with tubulin (POD) to a compound that blocks the topoisomerases II action (VP-16), could be affected [15,17].

In the present study, we explored the cytotoxic effects of lignans **1**–**4** on the breast cancer cell line MCF-7, which is an ER-positive/PgR-positive luminal mammary carcinoma, endocrine responsive and often chemotherapy responsive. These compounds were also tested on the breast cancer (BC) cell line MDA-MB-231, characterized as a triple-negative/basal-B mammary invasive ductal adenocarcinoma, and the BC cell line BT-549, which is a triple-negative/basal-B mammary papillary invasive ductal carcinoma, and a mucin producer type. These triple negative cell lines possess an intermediate response to chemotherapy [3,18,19]. Triple negative breast cancer (TNBC) cells represent one of the most aggressive and difficult to treat subtypes of human breast cancer. Moreover, their ability to breach the basement membrane of the epithelial barrier and migrate distinguishes highly metastatic TNBC cells from non-metastatic breast cancer cells. The molecular basis for the aggressiveness of TNBC are largely unknown and yet to be clarified [20,21,22].

Compounds **1**, **2**, and **4** were previously isolated and characterized by Rojas-Sepulveda and collaborators and Antúnez and collaborators in 2012 and 2016 respectively [23,24]. Briefly, the compounds, namely 5′-demethoxy-β-peltatin-A-methylether (**1**), acetylpodophyllotoxin (**2**), 5′-demethoxydeoxypodophyllotoxin (**3**), and 7′,8′-dehydroacetylpodophyllotoxin (**4**), were isolated through a bioactivity-directed study of the stem bark of *Bursera* *fagaroides* var. *fagaroides* (Burseraceae) through chromatographic methods and by 1D- and 2D-NMR, as well as FAB-MS analyses (Figure 1). Both the Rojas-Sepulveda and Antúnez research teams reported the cytotoxic potential of compounds **1**, **2**, and **4** against four human carcinoma cell lines (nasopharyngeal, KB; colon, HF-6; breast, MCF-7; and prostate, PC-3). Based on these preliminary results, this work continues with the cytotoxic study of compounds **1**, **2**, and **4**, in addition with the not previously evaluated compound **3**, focusing specifically against breast cancer. Furthermore, these compounds were evaluated in their clonogenicity and selectivity against three BC cell lines: MCF-7, MDA-MB-231, and BT-549, as well as the non-tumorigenic mammary epithelial cell line MCF-10A.

## 2. Results

### 2.1. Cytotoxicity and Clonogenicity Assays, and the Selctive-index

**Figure 2:**
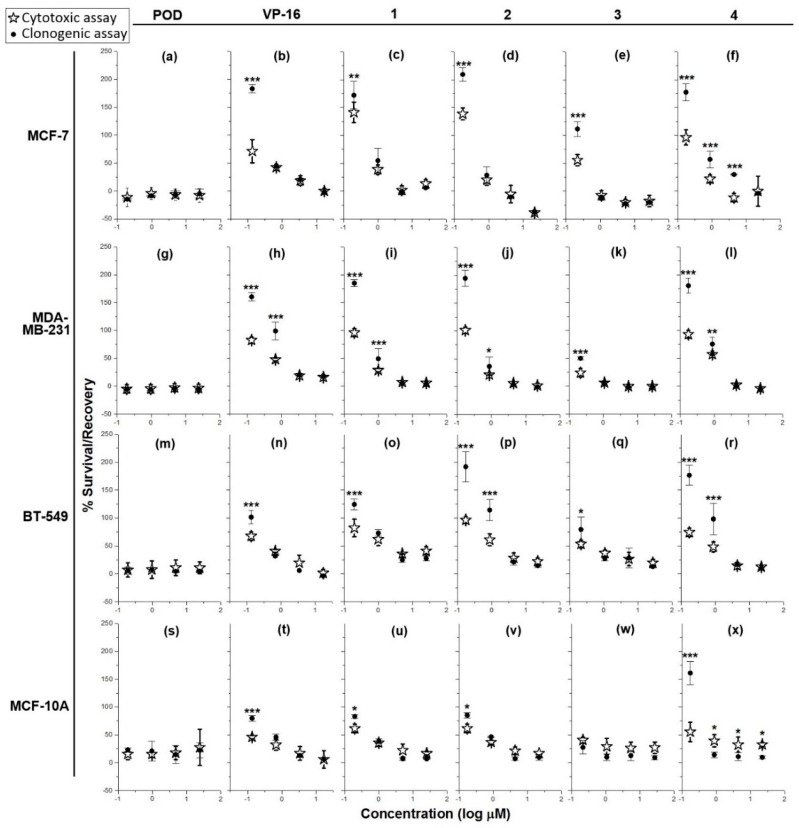
Graphs showing cell survival rates in cytotoxic assay after 72 h of treatment (✰) with controls (POD and VP-16) and lignans (compounds **1**–**4**), and recovery rates in clonogenic assay (●) (cytocidal and/or cytostatic activity) after 72 h post-treatment against four cell lines: MCF-7 (**a**–**f**), MDA-MB-231 (**g**–**l**), BT-549 (**m**–**r**), and MCF-10A (**s**–**x**) (*n* = 3). Percentages of survival and recovery were compared with a one-way ANOVA and Bonferroni correction (* = *p* < 0.05, ** = *p* < 0.01, and *** = *p* < 0.001).

Treatment of MCF-7, MDA-MB-231, BT-549, and MCF-10A (Figure 2a,g,m,s, respectively) with different POD concentrations resulted in the arrest of cell proliferation, which persisted even after the toxic agent was removed.

On the other hand, treatment of MCF-7, BT-549, and MCF-10A with VP-16 (Figure 2b,n,t, respectively) resulted in a concentration-dependent effect. Cells treated with 10, 2, and 0.4 µg/mL did not recover proliferation after treatment, as shown by the lack of significant difference (*p* < 0.05) between the percentage of survival and the percentage of recovery. However, VP-16 at 0.08 µg/mL showed a significant difference between the percentage of survival (MCF-7 = 71% ± 20.7%; BT-549 = 67% ± 7.5%; MCF-10A = 46% ± 2.8%), and the percentage of recovery (MCF-7 = 183% ± 7.4%; BT-549 = 101% ± 11.7%; MCF-10A = 79% ± 5.1%). In the MDA-MB-231 cell line, VP-16 (Figure 2h) displayed dose-dependent behavior but only higher concentrations (10 and 2 µg/mL) caused inhibition of proliferation and cell recovery in clonogenic assay; treatments with 0.4 and 0.08 µg/mL of VP-16 presented a survival of48% ± 2.4% and 82% ± 5.6% respectively, and a recovery rate of 98% ± 16.1% and 160% ± 7.4% respectively.

Addition of compound **1** to MCF-7, BT-549, and MCF-10A cell lines (Figure 2c,o,u, respectively showed a dose-dependent cytotoxic effect at concentrations of 10, 2, and 0.4 µg/mL, which persisted after removal of the treatment, as inferred by the lack of statistical significance between the cytotoxic and cytostatic data. The percentage of survival of cells treated with the lowest concentration (0.08 µg/mL) of compound **1** was as follows: MCF-7 = 130% ± 18.55%; BT-549 = 82% ± 15.7%; and MCF-10A = 61% ± 7.9%. On the other hand, the percentage of recovery was: MCF-7 = 172% ± 24.5%; BT-549 = 124% ± 9.7%; and MCF-10A = 83% ± 3.3%. In the MDA-MB-231 cell line (Figure 2i), concentrations of 10 and 2 µg/mL inhibited proliferation and blocked recovery, while treatment with 0.4 and 0.08 µg/mL of compound **1** resulted in a percentage of survival of 28% ± 3.1% and 96% ± 6.9% respectively, and a percentage of recovery of 49% ± 18.6% and 184% ± 6.4% respectively.

Addition of compound **2** to MCF-7 and MCF-10A (concentrations of 10, 2, and 0.4 µg/mL) dose-dependently inhibited the proliferation and the recovery of cells (Figure 2d,v). However, treatment with 0.08 µg/mL resulted in a 121% ± 10.8% of survival and 209% ± 12.4% of recovery in MCF-7 cells and a 61% ± 7.8% of survival and 85% ± 4.5% of recovery in MCF-10A cells. Treatment of MDA-MB-231 and BT-549 (Figure 2j,p) with 10 and 2 µg/mL of compound **2** completely inhibited proliferation and prevented recovery. Conversely, addition of 0.4 and 0.08 µg/mL to MDA-MB-231 resulted in a survival rate of 21% ± 0.6% and 101% ± 5.7% respectively. Addition of the same concentrations to BT-549 cells resulted in a survival rate of 61% ± 10.6% and 96% ± 5.5% respectively. The percentage of recovery of MDA-MB-231 treated with 0.4 and 0.8 µg/mL was 36% ± 16.8% and 194% ± 14.7% respectively; the recovery rate of BT-549 at the same concentrations was around 114% ± 18.5% and 191% ± 27.1% respectively.

Compound **3** was the most toxic of the tested lignans. Addition of 10, 2, and 0.4 µg/mL on MCF-7, MDA-MB-231, and BT-549 cells (Figure 2e,k,q) resulted in cytotoxicity with a lack of recovery; treatment of MCF-7 with 0.08 µg/mL of compound **3** resulted in a survival rate of 55% ± 9.9% and a recovery rate of 111% ± 13%. In the case of the MCF-10A cell line, the four tested concentrations of compound **3** completely inhibited proliferation in the cytotoxicity and clonogenicity assays (Figure 2w).

Tested on MCF-7, compound **4** (Figure 2f) at the highest concentration (10 µg/mL) did not exhibited differences between percentages of survival and recovery but at lower concentrations a dose-dependent activity was observed. Indeed, at 2, 0.4, and 0.08 µg/mL, the survival rate was 11% ± 7.6%, 21% ± 7.2%, and 96% ± 13.5%, while the recovery rate was 29% ± 0.7%, 57% ± 14.5%, and 177% ± 15.6%, respectively. Addition of compound **4** to MDA-MB-231 and BT-549 (Figure 2l,r), also showed an inhibitory effect on the survival and recovery at concentrations of 10 and 2 µg/mL; however, this was not the case when lower concentrations were added. In the latter case, the percentages of survival obtained at 0.4 and 0.08 µg/mL were: MDA-MB-231 = 57% ± 1.4% and 93% ± 6.9%; BT-549 = 48% ± 8.8% and 74% ± 7.4% respectively. At the same concentrations, the percentages of recovery were: MDA-MB-231 = 75% ± 12.2% and 180% ± 13.5%; BT-549 = 98% ± 27.9% and 176% ± 18% respectively). In the MCF-10A control cell line, compound **4** (10, 2, and 0.4 µg/mL) exhibited a concentration-independent effect (Figure 2x). However, while there were statistically significant differences (*p* < 0.05), these were the result of a decrease in the % of recovery compared with the % of survival. At the lowest concentration (0.08 µg/mL), the % of survival was about 55% ± 17.2%, while the % of recovery was 160% ± 21%.

**Figure 3:**
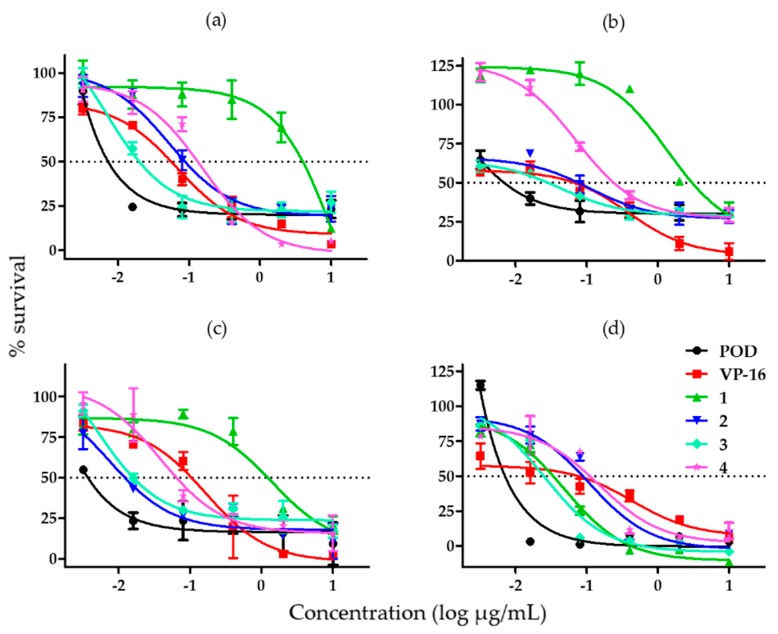
Graphs showing cell survival rates obtained by adding different concentrations of lignans from *B. fagaroides* var. *fagaroides* to (**a**) MCF-7 cells; (**b**) MDA-MB-231 cells; (**c**) BT-549 cells; and (**d**) MCF-10A cells (*n* = 3).

With the aim of comparing the effects of compounds **1**–**4** and controls, their half inhibitory concentration (IC_50_) value was calculated. The cytotoxic activity assay was repeated in order to obtain a larger cytotoxicity spectrum (Figure 3).

When we tested the effect of each compound on the MCF-7 cell line, we found that compound **3** was the most potent and compound **1** was the least potent amongst the substances tested against MCF-7 (Figure 3a). Figure 3b shows the effects of the compounds against the MDA-MB-231 cell line, which were similar to the ones obtained for MCF-7. Compound **2** was the most potent against BT-594, but its effect was comparable to that of compound **3**; compound **1** was once more the least potent (Figure 3c). In Figure 3d, showing the effects of the compounds in MCF-10A cells, we can appreciate that compounds **1** and **3** have a similar high potency, just as compounds **2** and **4** have a similar low potency.

Table 1 summarizes the estimated IC_50_ values of all compounds analyzed in this study on the cancer cell lines MCF-7, MDA-MB-231, and BT-549, and on the non-tumorigenic immortalized cell line MCF-10A. Compound **1** showed the highest IC_50_, indicating a low potency against all cancer cell lines (MCF-7 = 7.22 ± 0.09 µM; MDA-MB-231 = 2.44 ± 0.08 µM; and BT-549 = 1.26 ± 0.08 µM). Compound **3** showed the lowest IC_50_ against MCF-7 = 0.04 ± 0.01 µM; MDA-MB-231 = 0.145 ± 0.04 µM; and MCF-10A = 0.09 ± 0.009 µM. However, the IC_50_ of compounds **1**, **2**, and **3** on BT-549 cells were comparable. As shown in Table 1 POD presented a lower IC_50_ than VP-16 in all cell lines.

Table 2 summarizes the results of the comparison between potencies of compounds **1**–**4** with positive controls. We observed that compounds **2**, **3**, and **4** had higher potency than VP-16 on the BT-549 cell line (14.1, 7.6, and 2.6 respectively); their potency on the other cell lines was comparable with that of either POD or VP-16.

The immortalized human mammary epithelial cell line MCF-10A, was used as a control in the selectivity analysis due to its non-tumorigenic origin [25]. The in vitro activity of the tested compounds on the MCF-10A cell line was used to estimate the selectivity index with the formula described in the Materials and Methods section (Equation (2)). As shown in Table 3, compounds **2** and **4** presented the best selectivity index against the BT-549 cell line (28.1 and 7.43 respectively). However, according to the “selectivity criteria” only compound **2** could be considered a selective compound against BT-549. Compounds **3**, **4**, and POD showed a selectivity index < 10 on BT-549 cell line. Compounds with SIs lower than 10 but higher than 1 could be considered as non-selective.

### 2.2 Similarity Analysis of Compounds

Tanimoto coefficients (Tc) are summarized in Table 4. According to the used descriptors, and the binary strings analysis (Tables S1 and S2), all the analyzed compounds have greater similarity to POD (Tc > 0.38 but < 0.50) than to VP-16 (Tc > 0.18 but < 0.38). Compounds **2** and **3** have a Tc of 0.50 indicating a 50% structural similarity with POD, in contrast, compound **3** has a Tc of 0.38 when compared to VP-16. POD and VP-16 have a Tc of 0.33 when compared between them.

## 3. Discussion

### 3.1. Cytotoxicity and Clonogenicity Assays, and the Selective-Index

The results comparing cytotoxicity and clonogenicity assays (Figure 2) showed that all tested compounds had dose-dependent activity, and both cytostatic and cytocidal activities against the breast cell lines tested treatment have been observed. Here, we have evaluated the capacity of lignans and controls to inhibit proliferation after 72 h of treatment, and determined if the duration and concentration were sufficient to stimulate cell death or senescence processes.

Podophyllotoxin (POD) is the most potent compound to inhibit cell growth of both tumor and non-tumor cells; furthermore, its effect is such that cells do not recover after treatment with this compound, even at the lowest concentration (0.08 µg/mL). VP-16 also showed an inhibitory effect on all cell lines; however, this effect was concentration-dependent. An effect on recovery was observed in the four breast cell lines when 0.08 µg/mL was used, suggesting that VP-16 may be acting as a cytostatic compound that inhibits cell growth when present at a medium to low concentration, but, when it is removed, would not affect the proliferation of the remaining cells.

Compounds **1** and **2** showed a concentration-dependent inhibitory effect. However, at concentrations of 10 and 2 µg/mL, the effect was so strong that it did not allow surviving cells to recover and proliferate (MCF-7 and MDA-MB-231). On the other hand, the concentration of 0.08 µg/mL had no effect on the inhibition of proliferation, since it was still at 100%; likewise, the recovery was very close to 200%, which corresponded to the value of proliferating cells in the presence of dimethyl sulfoxide (DMSO) alone (vehicle). Compound **3** had a higher effect since it inhibited cell proliferation at 10, 2, and 0.4 µg/mL, and cell recovery after treatment with this compound was not observed. Compound **4** is a substance with low water solubility and consequently, after treatment, it was found to have partially crystallized in the culture medium. However, low concentrations (0.4 and 0.08 µg/mL) of **4** had a dose-dependent cytostatic effect in the four cell lines tested; on the other hand, higher concentrations (2 and 10 µg/mL) promoted cytocidal activity. In all cases, inhibition of proliferation and deficit in recovering could result in cell death or cellular senescence, which did not involve protein degradation, as seen from the SRB assay quantification results. Compounds **3** and **4** showed an inverse percentage of recovery compared to the percentage of survival in the MCF-10A cell line (Figure 2w,x), suggesting that this could be the result of a cytotoxic effect that needed a longer time to display low protein levels.

Treatment with POD resulted in an interesting effect on cancer cell lines. MCF-10A cell treated with POD showed about 20% survival even at high concentrations (10, 2, 0.4, 0.08, and 0.016 µg/mL), and did not reach zero survival. Possible explanations for this may be because degradation of proteinaceous material had not yet been initiated, or that the cells were not subjected to cell death involving protein degradation. In the literature, POD has been considered a very toxic compound when administrated in vitro and in vivo, and that was observed in our results, as POD presented the highest potency compared to lignans or VP-16 (Table 2). Conversely, POD demonstrated non-selective activity in our selectivity analysis.

Treatment of MCF-7, MDA-MB-231, BT-549, and MCF-10A cells with VP-16 showed a direct dose-dependent effect. The MDA-MB-231 cell line was the most sensitive to VP-16 treatment, which was a non-selective compound according to analysis of selectivity.

Based on testing in cancer cell lines, compound **1** presented the lowest cancer cell proliferation inhibition activity. A high concentration of **1** was needed to obtain half inhibition (MCF-7 = 7.2 µM; MDA-MB-231 = 2.4 µM; and BT-549 = 1.2 µM). In contrast, a low concentration of compound **1** (0.13 µM) was needed to obtain half inhibition in MCF-10A cells.

Compound **2** is the most promising for continued research in BT-549 cells, due to its potency of 14.1 times stronger when compared with VP-16, but also for its selectivity index of about 28.1 in the same cell line. As shown in Figure 3a–c, the compound **2** activity spectrum could be considered similar to that of POD, specifically at the highest concentrations where survival did not reach zero. It is important to remember that BT-549 is a triple negative breast cancer (TNBC) cell line and represents one of the most aggressive and difficult to treat subtypes of human breast cancer.

Results from testing on MCF-7, MDA-MB-231, and MCF-10A cell lines showed that compound **3** was the most potent. However, it exhibited potency comparable to that of compound **2** (Figure 3c) in the BT-549 cell line. Compound **3** is a non-selective compound (SI = 4.4) in the BT-549 cell line. Likewise, compound **3** exhibited an activity spectrum similar to that of POD.

Finally, compound **4** presented high activity (IC_50_) in cancer cells (MCF-7 = 0.35 µM; MDA-MB-231 = 0.16 µM; and BT-549 = 0.46 µM). When compared with VP-16, compound **4** was 2.6-fold more potent in the BT-549 cell line, and showed an SI value of 7.6.

Cells are not all affected in similar ways by different compounds, and that was observed in our results, despite the fact that the chemical structures of our compounds were comparable. Cancer cell lines have a resistance to death. For example, the MCF-7 cell line obtained from the American Type Culture Collection (ATCC) does not express the caspase-3 protein [26], and therefore cannot activate apoptosis by that downstream pathway. However, MCF-7 could potentially activate other cell death mechanisms. The MDA-MB-231 and BT-549 cell lines have altered p53 activity, which in normal conditions is activated in response to DNA damage [27]. POD, as a tubulin polymerization inhibitor [11], leads to cellular arrest, but our results indicate that the cells could have rendered senescent due to cytotoxic treatment. Although senescent cells do not proliferate, they remain metabolically active [28].

In several different studies, acetylpodophyllotoxin (**2**) has been part of quantitative high-throughput screening (qHTS) experiments in the cell line DT40, where it was found to have inhibitory activity against the repairing human protein tyrosyl-DNA phosphodiesterase 1 (TDP1), which has been proposed as a new target for anticancer drug development. TDP1 is not an essential protein, but during treatment with a topoisomerase I inhibitor (e.g., camptothecin), TDP1 was a critical factor for cell survival [29]. In contrast, it has been found that **2** interferes in the internalization process in a concentration-response assay for anthrax lethal toxin in a qHTS test in ME-180 cells [30]. The 5′-demethoxydeoxypodophyllotoxin compound (**3**), also called morelensin, has been part of studies associated with in vivo anticancer drug screening against intraperitoneal inoculation of melanoma (B16 cell line) and leukemia (L1210 cell line). However, subcutaneous inoculation in a model of Lewis lung carcinoma did not show biological activities [31].

### 3.2 Similarity Analysis of Compounds

According to the coefficients obtained with the Tanimoto equation (Table 4), compounds **2** and **3** shared 50% structural similarity with POD, while compound **4** had the lowest similarity. However, all the structures contained the five rings (A, B, C, D, and E, and therefore exhibited the characteristic cytotoxic potential of the podophyllotoxin type lignans. It has been suggested that higher or lower potency derives from the structural changes present in the molecules, which are closely related to their conformer at their site of action. Martin and collaborators demonstrated in 2002 [32] that structurally similar compounds have similar biological activity, and that as the structural similarity increases, the biological similarity increases as well. Nevertheless, despite the fact that the resemblance of compounds and controls is very high, their mechanism of action are distinct. According to the literature, lignan compounds (podophyllotoxins) isolated from *B. fagaroides* var. *fagaroides* have the structural ability to act as tubulin inhibitors mainly because of the C-7 α configuration; the missing methoxy group in C-3′ (ring E) in compounds **1** and **3** does not change their in vitro activities. The C-6 methoxy addition in **1** and the acetyl group in C-7 in **2** are essential for the inhibition of the mitotic spindle. Furthermore, the double bond between C-7′ and C-8′ in compound **4** does not affect its anti-tubulin activity [10,16,33]. Unlike podophyllotoxins, epipodophyllotoxins like VP-16 do not affect microtubule assembly and do not arrest cells in mitosis, but rather exert their maximal effects later, in the S or G_2_ phase, and prevent cells from entering mitosis [34]. Beers and collaborators, in 1988 [35], concluded that a non-aromatized ring C is structurally required for the potent inhibitory activity against topoisomerase II, and that a free OH at C-4’ contributes to significant cytotoxicity.

Controls used were selected due to structural similarities among them (both lignan type compounds with A, B, C, D, and E rings). However, according to the Tanimoto coefficient (using our descriptors) they presented 33% similitude. POD and VP-16 have no usefulness in clinics against breast cancer. Nevertheless, we have continued research of compounds **1** to **4** owing to preliminary results against MCF-7 and their low solubility in water (lipophilicity). All these compounds could be used in breast cancer treatment, considering that among the many different cell types surrounding breast cancer cells, the most abundant are those comprising mammary adipose tissue [36].

Several of these compounds known as podophyllotoxins have been isolated from plants, and have been the starting point for subsequent structural changes. Nevertheless, only epipodophyllotoxins (etoposide and teniposide) have been used in the clinic as anticancer agents. However, treatment failure due to the increased resistance of tumor cells, and adverse effects caused by treatment, have prompted the search for therapeutic alternatives. Therefore, it is very important to find bioactive and selective compounds in order to ameliorate patient conditions, specifically during adverse effects associated to the chemotherapy, in addition to bioactive compounds that could prevent tumor cell proliferation.

## 4. Materials and Methods

The compounds (**1**–**4**) used in this study were isolated and fractionated by Laura Alvarez-Berber. Spectroscopic, spectrometric, and purity data of compounds **1**, **2**, and **4** have been previously reported by Alvarez-Berber and collaborators [23,24,37]. 5′-desmethoxydesoxypodophyllotoxin (**3**), also known as morelensin, was obtained by chromatographic methods by Álvarez-Berber and collaborators from the active fraction two (F-2) in their previously described work [23,37]. Compound **3** was obtained as a yellowish amorphous powder, mp 180–182 °C; ${\left[\alpha \right]}_{D}^{24}$ −126.0° (*c* 0.014, CHCl_3_). ^1^H-RMN (400 MHz, CDCl_3_), δ ppm: 6.55 (s, 1H), 6.51 (s, 1H), 2.2 (dd, 1H, *J* = 8 Hz), 2.3 (dd, 1H, *J* = 7.2 Hz), 2.6 (m, 2H), 4.01 (m, 2H, *J* = 16 Hz), 4.14 (m, 2H, *J* = 15.8 Hz), 6.75 (s, 1H), 6.68 (d, 1H, *J* = 8 Hz), 6.5, (s 1H), 4.2 (d, 1H, *J* = 2 Hz), 2.60 (m, 2H), 5.92 (s, 2H), 3.81 (s, 3H), 3.85 (s, 3H). ^13^C-RMN (100 MHz CDCl_3_), δ ppm: 128.02 (C-1), 129.81 (C-2), 109.23 (C-3), 146.64 (C-4), 146.95 (C-5), 121.76 (C-6), 32.08 (C-7), 34.4 (C-8), 64.17 (C-9), 129.73 (C-1′), 111.07 (C-2′), 149.43 (C-3′), 148.03 (C-4′), 108.24 (C-5′), 111.96 (C-6′), 62.5 (C-7′), 33.91 (C-8′), 174.88 (C-9′), 105.258 (O-CH_2_-O), 55.95 (CH_3_O-C-3′), 55.95 (CH_3_O-C-4′). FABHRMS *m*/*z* 370 [M + H]^+^ (calcd for C_21_H_20_O_6_, [M + H]^+^ 368.38). These data match the physicochemical and spectroscopic data from the literature [38,39].

The MCF-7 cell line (HTB-22) was donated by Maria Luisa Villarreal-Ortega; cell lines MDA-MB-231 (ATCC: HTB-26), BT-549 (ATCC: HTB-122), and MCF-10A (ATCC: CRL-10317) were purchased from the American Type Culture Collection (ATCC, Manassas, VA, USA). Cancer cells cultures were grown in RPMI-1640 medium (Sigma-Aldrich, Saint Louis, MO, USA) supplemented with: 10% fetal bovine serum (Mexico origin, USDA approved) (Biowest, Nualillé, France); 2 g/L NaHCO_3_ (Sigma-Aldrich, cat. S8761), and incubated in a 5% CO_2_ incubator at 37 °C; MCF-10A were cultured in MEGM medium (Lonza BulletKit, Basel, Switzerland) using the above incubating conditions. Cells were thawed according to the standard methods and a cell bank was developed for each cell line. All experiments were performed using freshly thawed cells after three passages.

### 4.1. Cytotoxicity and Clonogenicity Assays

In vitro cytotoxicity and clonogenicity assays were performed using the sulforhodamine B (SRB) (MP Biomedicals, LLC) protein staining assay [40,41,42,43], using the cell lines described before. Briefly, cells were seeded in 384- or 96-well microtiter plates at a density of 1 × 10^4^ cells/mL, and placed in an incubator (5% CO_2_ and 37 °C) for 8 h. Afterward, different concentrations (0.08, 0.4, 2, and 10 µg/mL) of pure compounds and positive controls (POD and VP-16) were added in triplicate and incubated for 72 h.

Afterward, cells were fixed with cold trichloroacetic acid (30% in water) and stained with SRB (0.4% in a 1% of acetic acid solution). Cells were washed with 1% of acetic acid solution. Finally, the bound colorant was solubilized with Tris-base to obtain optical density (OD_sample_). The bound colorant was proportional with either total protein or cells amount. DMSO (final concentration of 0.5%) was used as vehicle and blank (OD_blank_). The total protein concentration in a single plate with cells at the beginning of the assay was considered as zero (OD_zero_). Microtiter plates were incubated for 72 h after which the total protein concentration was determined with Equation (1). This assay measures the respective absorbance at 490 nm using a spectrophotometer (Molecular Devices, SPECTRA max plus 384).

% survival/proliferation = (OD_sample_ − OD_zero_)/(OD_blank_ − OD_zero_) × 100 (1)

To determine the recovery percentage, an in vitro clonogenicity assay was performed. Cells were seeded, incubated, and treated in microtiter plates under the previously described conditions. After 72 h of treatment, the culture media of each well was removed, followed by two washes with phosphate buffered saline (PBS), before warm media supplemented with FBS was added to the cells and incubated for an additional 72 h in the same conditions. The percentage of proliferation was then determined using Equation (1).

**Figure 4:**
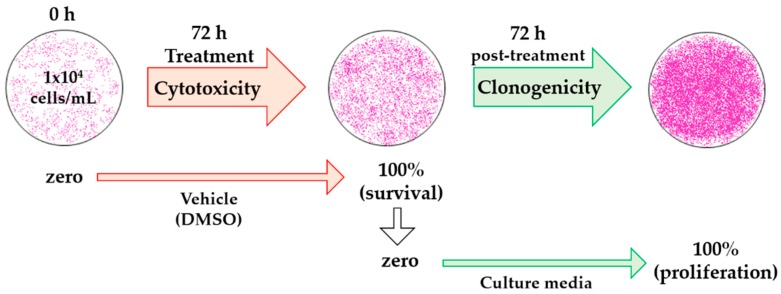
Schematic diagram of the analysis with dimethyl sulfoxide (DMSO) treatment for cytotoxic and clonogenic determinations. The optical density (OD) of initially plated cells was measured as zero (OD_zero_) in the cytotoxicity assay, OD after 72 h of treatment with vehicle was considered as 100% cell survival (OD_blank_). In the clonogenic assay, OD at 100% cell survival was considered as zero, since these were the initial cells (OD_zero_), and OD at 72 h vehicle post-treatment was measured as 100% cell proliferation (OD_blank_).

We have defined percentage of recovery as the percentage of survival at 72 h of treatment added to the percentage of proliferation calculated 72 h post-treatment. This is outlined in Equation (2) and Figure 4:

% recovery = % survival (cytotoxicity assay) + % proliferation (clonogenicity assay)(2)

The half inhibitory concentrations of lignans and controls were calculated from six concentrations (0.0032, 0.016, 0.08, 0.4, 2, and 10 µg/mL). Data analysis was performed using the relationship between the % of survival cells (total protein, Equation (1)) and the relative optical density vs. concentration as a logarithm expression. The IC_50_ values were reported as a mean of three independent experiments ± standard deviation (S.D.).

### 4.2. Selective-Index

To determine the cytotoxic selectivity of the substances tested, the selectivity index (SI) was calculated according to the following equation:

SI = IC_50_^no cancer cells^/IC_50_^cancer cells^ (3)

where a SI ≥ 10 was considered to belong to a selective compound, according to Quispe and collaborators [44] and Valdés-García and collaborators [45].

### 4.3. Similarity Analysis of Compounds

The similarity among compounds was obtained manually in an Excel sheet, employing the Tanimoto coefficient (Tc); we used fifteen structural fragments as molecular descriptors (available in the supplementary material); the Tc of each compound was analyzed and compared to that of POD and VP-16. The Tc ranges remain between 0.00 and 1.00, where 0.00 indicates no similarity and 1.00 indicates 100% similarity with the reference [46,47].

The equation for the Tanimoto coefficient is:

Tc = c/(a + b − c)(3)

where a—Bits set in reference structure. b—Bits set in enquiry structure. c—Bits set in common between the reference structure and enquiry structure.

### 4.4. Statistical Analysis

To analyze the difference between the cytotoxic effect and recovery, the averages in the percentage of survival and percentage of recovery were compared with a one-way ANOVA and Bonferroni correction, using the statistical program GraphPad Prism, Version 5.00 (GraphPad Software, Inc., La Jolla, CA, USA).

Graphs were obtained using the relationship between the logarithm of the molar concentration vs. the percentage of survival; the curves were analyzed with a mathematical sigmoid model and the IC_50_ was determined by a sigmoidal fit with the function of Boltzmann. VP-16 results were analyzed by fitting a straight line and performing a linear regression to determine the IC_50_; both analyses were performed using OriginPro version 9.0.0 (OriginLab Corp., Northampton, MA, USA). For all statistical tests, significance was established at *p* < 0.05.

## 5. Conclusions

In conclusion, the lignans (**1**–**4**) evaluated in this study isolated from *B. fagaroides* var. *fagaroides* demonstrated cytotoxic (IC_50_ range = 0.011–7.22 µM) and cytocidal (≥0.08 µg/mL) activity against the breast cell lines MCF-7, MDA-MB-231, BT-549, and MCF-10A. Compounds **2** and **3** were more potent (14.1 and 7.6 respectively) than VP-16 in the BT-549 cell line.

The in vitro activity of acetylpodophyllotoxin (**2**) indicate a selective activity on BT-549 (SI = 28.17), a TNBC type of cell line, that represent one of the most aggressive and difficult to treat subtypes of human breast cancer. Hence, this preliminary result suggests that future experiments are indeed required.

Structural similarity of the compounds, according to Tc, suggested a greater similarity with podophyllotoxin structure than that of VP-16, suggesting a possible mechanism of action through the inhibition of the mitotic spindle.

## Figures and Tables

**Table 1:** Cytotoxic activity (IC_50_ µM) of lignans. IC_50_ values are mean of three independent experiments ± S.D.

Compounds	MCF-7	MDA-MB-231	BT-549	MCF-10A
**1**	7.222 ± 0.098	2.444 ± 0.087	1.269 ± 0.087	0.137 ± 0.015
**2**	0.132 ± 0.017	0.180 ± 0.003	0.011 ± 0.004	0.318 ± 0.012
**3**	0.040 ± 0.011	0.145 ± 0.045	0.021 ± 0.003	0.092 ± 0.009
**4**	0.353 ± 0.088	0.161 ± 0.024	0.061 ± 0.012	0.468 ± 0.002
POD	0.018 ± 0.001	0.024 ± 0.006	0.005 ± 0.001	0.020 ± 0.008
VP-16	0.124 ± 0.026	0.038 ± 0.004	0.160 ± 0.010	0.047 ± 0.009

**Table 2:** Potency of compounds (**1**–**4**) compared to controls (POD and VP-16).

Cell Line	Controls	1	2	3	4
MCF-7	POD	0.002	0.135	0.449	0.050
	VP-16	0.017	0.942	3.124	0.351
MDA-MB-231	POD	0.010	0.136	0.168	0.151
	VP-16	0.016	0.217	0.268	0.242
BT-549	POD	0.004	0.465	0.252	0.086
	VP-16	0.126	14.151	7.661	2.604
MCF-10A	POD	0.144	0.062	0.214	0.042
	VP-16	0.341	0.147	0.507	0.100

**Table 3:** Selectivity index of compounds (**1**–**4**) and controls (POD and VP-16).

Compounds	MCF-7	MDA-MB-231	BT-549
**1**	0.02	0.06	0.11
**2**	2.42	1.77	28.17
**3**	2.33	0.64	4.43
**4**	1.32	2.90	7.62
POD	1.11	0.81	3.76
VP-16	0.38	1.20	0.29

**Table 4:** Structural similarity coefficients (Tc). The compounds were compared against the controls, according to calculated coefficients.

Compounds	POD	VP-16
**1**	0.43	0.33
**2**	0.50	0.27
**3**	0.50	0.38
**4**	0.38	0.18
POD	1.00	0.33
VP-16	0.33	1.00

## Supplementary Materials

Supplementary materials can be accessed at: http://www.mdpi.com/1420-3049/21/8/1013/s1.

## Abbreviations

The following abbreviations are used in this manuscript:

SI: Selectivity index
WHO: World Health Organization
ER: Estrogen receptor
PgR: Progesterone receptor
Her2/neu: Human epidermal growth factor receptor 2
IUPAC: International Union of Pure and Applied Chemistry
NMR: Nuclear Magnetic Resonance
FAB-MS: Fast Bombardment-mass spectrometry
DSMO: Dimethyl sulfoxide
SRB: Sulforhodamine B
qHTS: quantitative High Throughput Screening
TDP1: Tyrosil-DNA-Phosphodiesterase 1
NIH: National Institutes of Health
NCI: National Cancer Institute
PBS: Phosphate Buffered Saline
ANOVA: Analysis Of Variance
MEGM: Mammary Epithelial Cell Growth Medium
RPMI: Roswell Park Memorial Institute medium
