# Supplementary material for: Cytotoxicity, Post-Treatment Recovery, and Selectivity Analysis of Naturally Occurring Podophyllotoxins from Bursera fagaroides var. fagaroides on Breast Cancer Cell Lines
Source: Molecules. 2016 Aug 4;21(8):1013. doi: 10.3390/molecules21081013 (PMC6274026; doi:10.3390/molecules21081013)
Supplement: Supplementary file 1 [file molecules-21-01013-s001.pdf]

Supplementary Materials: Cytotoxicity, Clonogenicity, and Selectivity Analysis of Naturally Occurring Podophyllotoxins from *Bursera fagaroides* var. f*agaroides* on Breast Cancer Cell Lines

Omar A. Peña-Morán, María L. Villarreal-Ortega, Laura Álvarez-Berber, Angélica Meneses-Acosta and Verónica Rodríguez-López

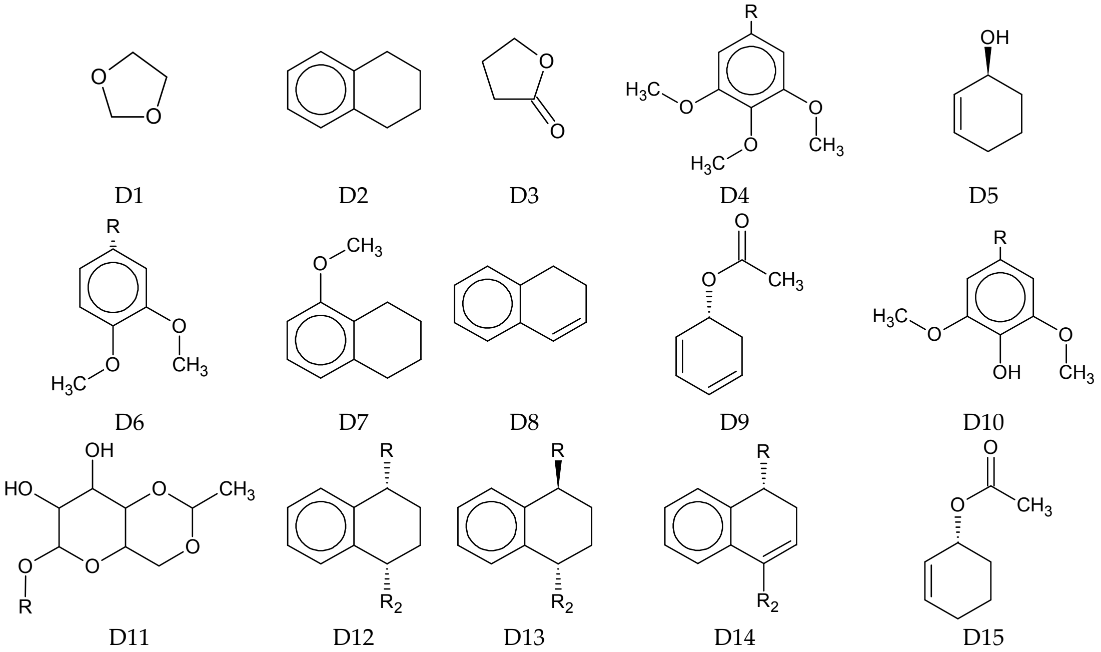

Figure S1. 2-D and 3-D molecular descriptors utilized in the analysis of similarity.

Table S1. Binary strings analysis from each compound and controls according to molecular descriptors.

Descriptors	POD	VP-16	1	2	3	4
D1	1	1	1	1	1	1
D2	1	1	1	1	1	0
D3	1	1	1	1	1	1
D4	1	0	0	1	0	1
D5	0	1	0	0	0	0
D6	0	0	1	0	1	0
D7	0	0	1	0	0	0
D8	0	0	0	0	0	1
D9	0	0	0	0	0	1
D10	0	1	0	0	0	0
D11	0	1	0	0	0	0
D12	1	0	0	1	0	0
D13	0	1	0	0	0	0
D14	0	0	0	0	0	1
D15	0	0	0	1	0	0
